# Exploring the Pharmacological Mechanism of the Herb Pair “HuangLian-GanJiang” against Colorectal Cancer Based on Network Pharmacology

**DOI:** 10.1155/2019/2735050

**Published:** 2019-11-29

**Authors:** Benjiao Gong, Yanlei Kao, Chenglin Zhang, Huishan Zhao, Fudong Sun, Zhaohua Gong

**Affiliations:** ^1^Affiliated Yantai Yuhuangding Hospital of Qingdao University, Yantai 264000, Shandong Province, China; ^2^Yantai Hospital of Traditional Chinese Medicine, Yantai 264000, Shandong Province, China

## Abstract

Since the herb pair Huang Lian-Gan Jiang (HL-GJ) was put forward as conventional compatibility for cold-heat regulation in the middle energizer in the theory of Traditional Chinese Medicine (TCM), their therapeutic effects were observed on the prevention and treatment of intestinal inflammation and tumors including colorectal cancer (CRC). However, the active compounds, crucial targets, and related pathways of HL-GJ against CRC remained unclear. The purpose of this research was to establish a comprehensive and systemic approach that could identify the active compounds, excavate crucial targets, and reveal anti-CRC mechanisms of HL-GJ against CRC based on network pharmacology. We used methods including chemical compound screening based on absorption, distribution, metabolism, and excretion (ADME), compound target prediction, CRC target collection, network construction and analysis, Gene Ontology (GO), and pathway analysis. In this study, eight main active compounds of HL-GJ were identified, including Gingerenone C, Isogingerenone B, 5,8-dihydroxy-2-(2-phenylethyl) Chromone, 2,3,4-trihydroxy-benzenepropanoic acid, 3,4-dihydroxyphenylethyl Alcohol Glucoside, 3-carboxy-4-hydroxy-phenoxy Glucoside, Moupinamide, and Obaculactone. HRAS, KRAS, PIK3CA, PDE5A, PPARG, TGFBR1, and TGFBR2 were identified as crucial targets of HL-GJ against CRC. There were mainly 500 biological processes and 70 molecular functions regulated during HL-GJ against CRC (*P* < 0.001). There were mainly 162 signaling pathways contributing to therapeutic effects (*P* < 0.001), the top 10 of which included DAP12 signaling, signaling by PDGF, signaling by EGFR, NGF signaling via TRKA from the plasma membrane, signaling by NGF, downstream signal transduction, DAP12 interactions, signaling by VEGF, signaling by FGFR3, and signaling by FGFR4. The study established a comprehensive and systematic paradigm to understand the pharmacological mechanisms of multiherb compatibility such as an herb pair, which might accelerate the development and modernization of TCM.

## 1. Introduction

Colorectal cancer (CRC) is the third major malignant tumor diagnosed globally and accounts for the fourth cancer mortality worldwide [1]. Furthermore, the incidence is still rising all over the world despite the major milestone in early diagnosis and treatment of CRC [2]. Clearly, it has become a powerful threat to public health due to high morbidity and mortality [3]. Although the pathogenesis of CRC is complex and still not fully illuminated, the interactions of risk factors including the environmental, lifestyle, and genetic factors play outstanding roles in initiation and ignition of CRC [4, 5]. The therapeutic regimens for CRC include surgery, chemotherapy, radiotherapy, immunotherapy, and targeted therapy [6–8]. The development of therapies for CRC still cannot cope with its high mortality owing to frequent recurrence and metastasis. Given this, it is in desperate need of more effective and less toxic treatment for CRC.

Traditional Chinese Medicine (TCM) has always played an important part in treating diseases for Asian people and is more and more widely recognized in western countries [9]. TCM has formed its own unique culture with differences in substance, methodology, and philosophy from modern medicine [10]. Multiherb compatibility has been regarded as the essence of TCM theories [11]. Herb pairs are the simplest and the most fundamental form of multiherb therapy and Chinese herb formulae often contain special herb pairs, which are asserted to assemble and interpret single combinations of traditionally classified herbal properties, connecting mutual enhancement, assistance, restraint and suppression, or antagonism [12]. Better pharmacological efficacy of herb pairs is usually due to the synergy effects from ingredients with special pharmacokinetic profile [13].

In TCM herbs, Huang Lian (HL) is derived from dried roots of *Coptis chinensis* Franch., *Coptis teeta* Wall., and *Coptis deltoidea* C. Y. Cheng et Hsiao, which are, respectively, called “Wei Lian,” “Yun Lian,” and “Ya Lian,” according to China Pharmacopoeia. Under the guidance of TCM theory, HL could alleviate heat, astringe extra fluids, and resolve toxin in the body. Zingiberis rhizoma (“Gan Jiang” in Chinese, GJ) is the dried root of *Zingiber officinale* Rocs distributed in Southwest China. GJ has the effects of warming the spleen and stomach for dispelling cold and restoring venation in accordance with China Pharmacopoeia. HL and GJ seem to be cold and hot in terms of medicinal properties and are not synergistic with each other. Since the creation of the herb pair “HL-GJ” for treating diseases of the spleen-stomach system by the ancient Chinese book “Treatise on Febrile Diseases,” combination of frigotherapy and pyretotherapy has become a conventional compatibility of cold-heat regulation in the middle energizer. Recent studies have found that the compatibility of HL with GJ could not only make their medicinal properties milder but also have strong synergistic effects and could increase pharmaceutical efficiency and reduce toxicity compared with individual applications. HL is a common medicine used to treat gastrointestinal diseases in the field of TCM. Modern pharmacological studies have shown that HL could inhibit invasion and metastasis of colorectal cancer cells and has inhibitory and clinically therapeutic effects on colon cancer [14, 15]. But HL often causes constipation, anorexia, and a series of symptoms of cold of insufficiency type due to its bitter and cold medicinal properties. Based on the theoretical guidance of combination of frigotherapy and pyretotherapy, compatibility of appropriate dose of GJ can alleviate these side effects of HL clinically, so that HL can take effect in expelling pathogenic factors and restoring the balance of human body. Chinese researchers have also reported that GJ can inhibit the proliferation and promote apoptosis of tumor cells. Although some achievements have been made in the pharmacological research studies of HL, GJ, and their monomeric substances, the studies on the molecular biology of the herb pair “HL-GJ” are relatively deficient. Hence, this study is expected to provide a theoretical basis for herb compatibility and achieve a breakthrough in the treatment of CRC.

Network pharmacology has been brought into focus in recent years, which integrates pharmacodynamics, pharmacokinetics, and system-level network analysis and can reveal the multifaceted mechanisms of herbal formulae treating complicated diseases from proteomics or at the systematic level [16–18]. Particularly, it has become a novel strategy to elucidate the interactive relationship between multicomponents and multitargets of TCM and a research hotspot to investigate multiple molecular mechanisms of multitarget compounds affecting biological networks for herbal medicines [19–21]. Therefore, we employed the network pharmacology to probe the pharmacological mechanisms of the herb pair “HL-GJ” against CRC in this study. Meanwhile, the relationships among herbs, compounds, and targets were also investigated. Finally, the multicompound, multitarget, and multipathway mechanisms were illuminated for HL-GJ against CRC based on network analysis.

## 2. Materials and Methods

### 2.1. Chemical Compounds of HL-GJ

Chemical compounds were obtained from the Traditional Chinese Medicine Systems Pharmacology Database [22] (TCMSP, http://ibts.hkbu.edu.hk/LSP/tcmsp.php) and the Traditional Chinese Medicine Integrated Database [23] (TCMID, http://www.megabionet.org/tcmid/). Compounds were screened according to predicted oral bioavailability (OB) and drug-likeness (DL) values and reserved if OB ≥ 30% and DL ≥ 0.18, which was a recommended criterion by the TCMSP database. The constituent compounds of HL-GJ were summarized for further research after removing duplication.

### 2.2. Target Fishing for HL-GJ

Target fishing was executed to investigate potential targets of constituent compounds of HL-GJ. PharmMapper [24] (http://lilab.ecust.edu.cn/pharmmapper/), an online server using the pharmacophore mapping approach for potential drug target identification, was employed to predict the potential protein targets based on 3D molecular structure. The 3D molecular structure files (.SDF) were obtained from the PubChem [25] (https://pubchem.ncbi.nlm.nih.gov/), a public repository for providing information of chemical compounds and their biological activities. Compounds without precise structural information cannot be predicted targets and were removed. Eventually, predicted protein targets were harvested with normalized fit score >0.9. The final target information was normalized via UniProt (https://www.uniprot.org/) [21].

### 2.3. CRC Targets

Different target information associated with CRC was collected from TTD (https://db.idrblab.org/ttd/) [26] and OMIM (http://www.omim.org/) [27] databases. CRC targets were retrieved after deleting duplicate data. Common targets of both CRC and the chemical compounds were considered potential targets.

### 2.4. Protein-Protein Interaction Data

The data of protein-protein interaction (PPI) were obtained from String [28] (https://string–db.org, ver 10.5), with species limited to “*Homo sapiens*” and the confidence score >0.9. String is a database of known and predicted protein-protein interactions, which defines PPI with confidence score ranges (low confidence: score < 0.4; medium: 0.4 < score < 0.7; high: 0.7 < score < 0.9; highest confidence: score > 0.9).

### 2.5. Network Construction

Network construction was visualized using Cytoscape [29] (version 3.2.1) as follows: (1) herb-compound, compound-compound target, herb-compound-compound target networks; (2) PPI network was established by linking common targets between CRC and chemical compounds and other human proteins that directly or indirectly interacted with common targets; (3) herb-compound-compound target-CRC target-PPI network. In the network, three topological parameters were calculated by NetworkAnalyzer [30], involving in Degree, Betweenness Centrality, and Closeness Centrality. Just the nodes with “Degree,” “Betweenness Centrality,” and “Closeness Centrality” larger than the corresponding median values were recognized as crucial nodes of HL-GJ against CRC.

### 2.6. Gene Ontology and Pathway Analysis

GO biological process and molecular function were analyzed based on GO database and carried out via the BINGO plug-in of Cytoscape. The pathway enrichment analysis was carried out via the Reactome FI plug-in based on the Reactome database. During these procedures, the threshold was set to 0.001, and *P* < 0.001 suggested statistical significance of the enrichment degree.

## 3. Results and Discussion

### 3.1. Herb-Compound-Compound Target Network

**Figure 1:**
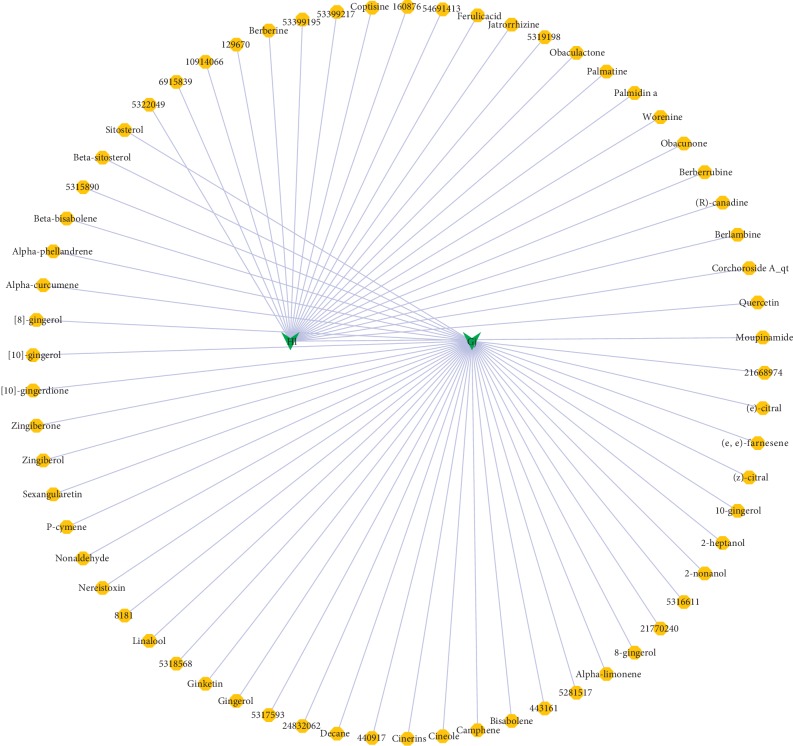
Herb-compound network (yellow octagons represented chemical compounds with oral bioavailability (OB) ≥30% and drug-likeness (DL) ≥0.18). Green arrow: herb; yellow octagon: chemical compound.

**Figure 2:**
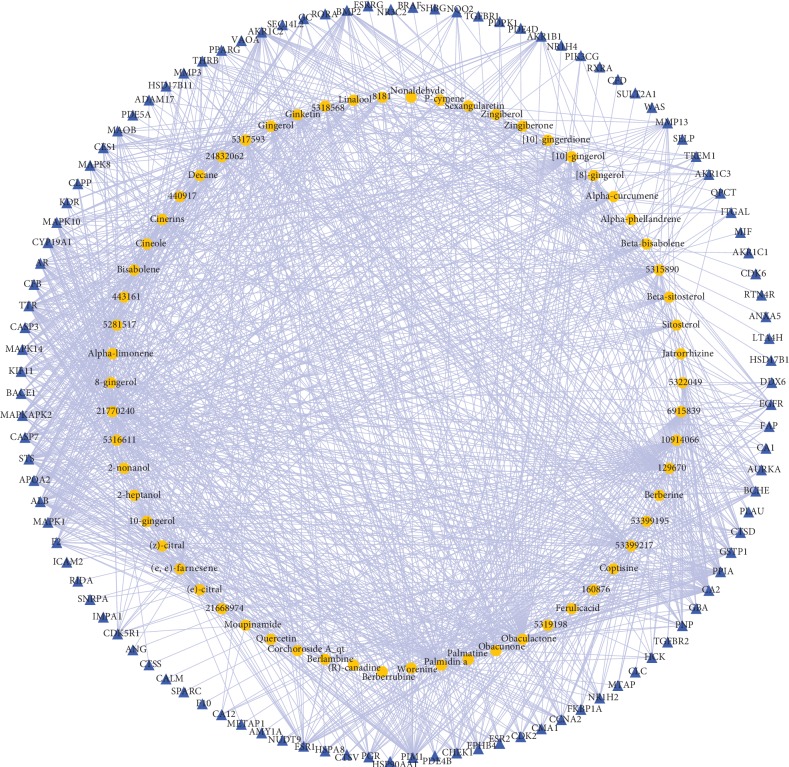
Compound-compound target network (blue triangles represented predicted protein targets with normalized fit score >0.9). Yellow octagon: chemical compound; blue triangle: chemical target.

**Figure 3:**
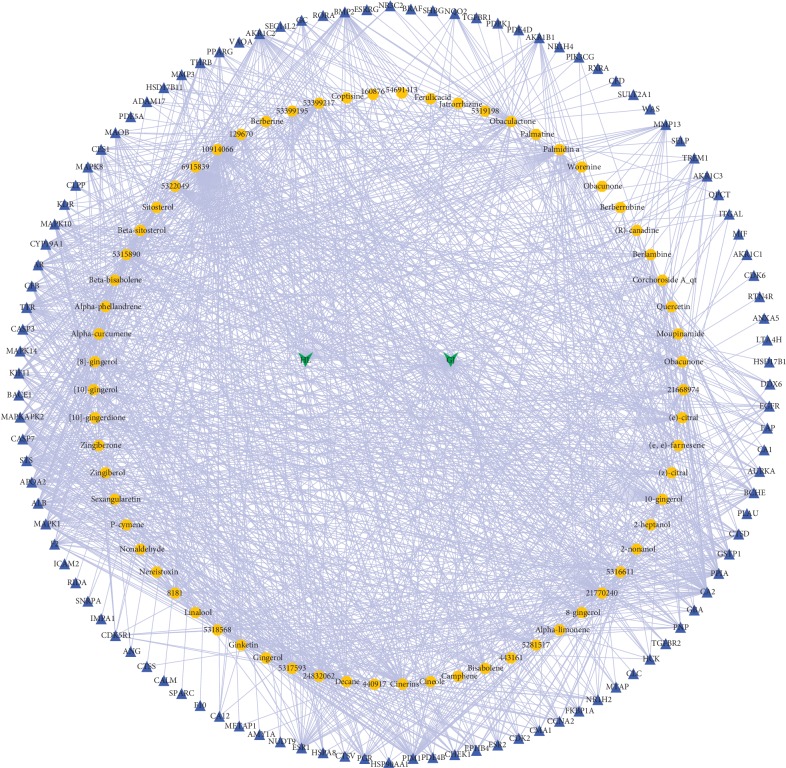
Herb-compound-compound target network integrated the relationship among herb, compound, and compound target, which might exert great influence during HL-GJ acting on CRC. Green arrow: herb; yellow octagon: chemical compound; blue triangle: chemical target.

As shown in Figure 1, the herb-compound network was composed of 67 nodes (2 herb nodes and 65 chemical compound nodes) and 65 edges. A total of 65 satisfactory chemical compounds were gained from the herb pair “HL-GJ,” including 24 in HL and 41 in GJ, which was consistent with the feature of multiple components of TCM (Tables [Supplementary-material supplementary-material-1] and [Supplementary-material supplementary-material-1]). Among the 65 chemical compounds, one compound could not be successfully predicted targets and two compound targets did not confirm to the filter criterion. So, the compound-compound target network contained 169 nodes (62 chemical compound nodes and 107 target nodes) and 1189 edges as shown in Figure 2 ([Supplementary-material supplementary-material-1]). In this network, it was not hard to find that each compound corresponded to multiple targets. For instance, Berberine in HL modulated PPIA, CA2, TTR, BCHE, AR, CYP19A1, and ESR2. Gingerol in GJ modulated 25 targets including PPIA, CA2, CCNA2, GSTP1, BCHE, MAOB, and so on. Also, PPIA was regulated by a number of compounds from HL and GJ. These phenomena were consistent with the feature of multiple targets of TCM and the synergy effect of multiherb compatibility.  Figure 3 integrated the herb-compound network and the compound-compound target network, which was convenient for observing the relationship among herb, compound and compound target, and the potential pharmacological effects of the herb pair “HL-GJ.” Overlong names of compounds were replaced with corresponding PubChem ID numbers in figures, which were summarized in Tables [Supplementary-material supplementary-material-1] and [Supplementary-material supplementary-material-1].

PharmMapper is widely employed for computational target detection and can offer top 300 potential targets for the query compound in default [31]. The predicted targets with a normalized fit score >0.9 were adopted in this study using PharmMapper. Several probable targets of active compounds from HL and GJ have been documented in other studies. Berberine can suppress AR signaling and present a promising mediator for the prevention or treatment of prostate cancer [32]. Chlorogenic acid may serve as a chemosensitizing mediator leading to tumor growth suppression due to its ability of activating or inhibiting some important pathways such as the EGFR/PI3K/mTOR pathway [33]. Columbianadin induced apoptosis of colon cancer (HCT116) cells, which was connected with the modulation of caspase-3, caspase-9, Bim, Bcl-2, Bax, and Bid [34]. Obacunone and obacunone glucoside (OG) induced the apoptosis of colon cancer (SW480) cells through reducing ratio of bcl2/bax gene transcription, activating caspase-3, and inducing fragmentation of DNA [35]. Quercetin might be an attractive chemical scaffold, which could generate novel derivatives such as PIM1, possessing various kinds of antikinase activities [36]. In 10-gingerol-treated human colon cancer (HCT116) cells, there was an increased ratio of Bax/Bcl-2 with induction of apoptosis through the activation of caspase-9, caspase-3, and ploy-ADP-ribose polymerase in a dose-dependent manner [37]. Active fractions including quercetin and β-sitosterol had an apoptotic effect on breast cancer (MCF-7 and MDA-MB-231) cells possibly through the mitochondrial pathway due to the activation of caspase3/7 [38]. The above description showed the precision of target prediction for PharmMapper.

### 3.2. PPI Network Analysis

**Figure 4:**
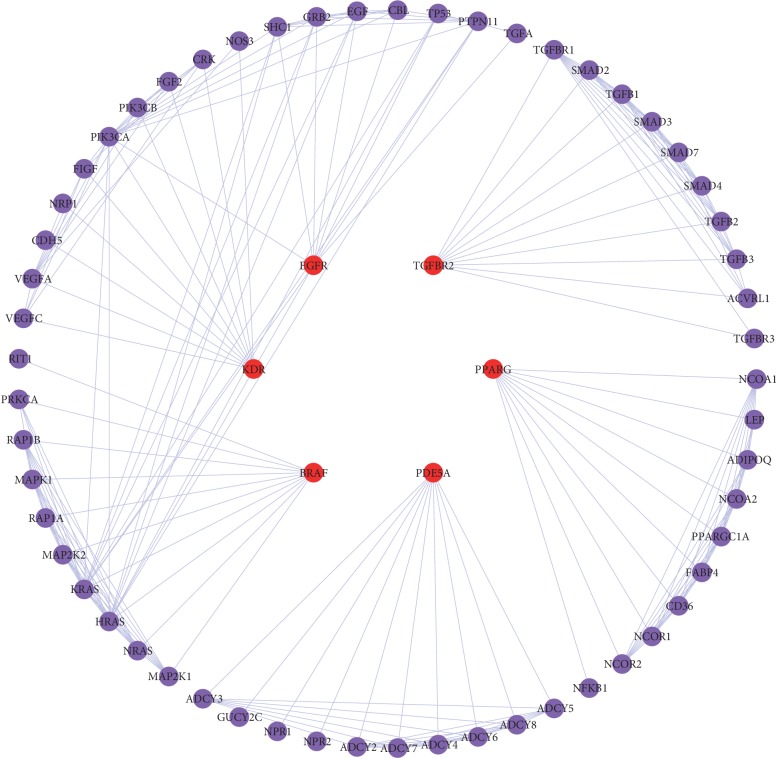
Protein-protein interaction network represented the reaction of HL-GJ response to CRC in vivo. Red ellipse: common target between CRC and chemical compounds; purple ellipse: human protein directly or indirectly interacted with common target.

One hundred and eighty-six CRC targets were collected from TTD and OMIM databases ([Supplementary-material supplementary-material-1]). Targets between CRC and chemical compounds were mapped, and 6 common targets were found. Fifty-seven other human proteins directly or indirectly interacted with 6 common targets were achieved from String database. The PPI network of the common targets is shown in Figure 4, including 63 nodes (6 common target nodes and 57 other human protein nodes), which might represent the reaction of HL-GJ response to CRC in vivo. NetworkAnalyzer was employed to calculate topological parameters such as Degree, Betweenness Centrality, and Closeness Centrality of the 63 targets in the PPI network ([Supplementary-material supplementary-material-1]) in order to identify key nodes in the network. The corresponding median values of Degree, Betweenness Centrality, and Closeness Centrality were 7.02, 0.04, and 0.63. Thus, the nodes with “Degree >7.02,” “Betweenness Centrality >0.04,” and “Closeness Centrality >0.63” were considered as key targets of HL-GJ against CRC. As a result, HRAS, KRAS, PIK3CA, PDE5A, PPARG, TGFBR1, and TGFBR2 were identified as crucial targets of HL-GJ against CRC.

RAS family members of proteins often appeared in mutated and oncogenic forms in human tumors. Four diverse RAS proteins were encoded by 3 genes: *KRAS* (2 splice variants), *HRAS*, and *NRAS* [39]. RAS protein mutations could result in nonreversible reduction in GTPase activity or inability of activating GTPase [40], and mutations in *KRAS* held about 85% of overall *RAS* mutations in human tumors; *NRAS* about 15%; and *HRAS* less than 1% [41]. The probability of *KRAS* mutation was approximately 30–50% in CRC [42], associated with advanced disease status, greater ratio of right-sided colon tumors, poor tumor differentiation, and more liver metastasis [43–45]. KRAS was also reported to be associated with mucin component and lymphovascular invasion [46]. KRAS was known to be an alternative marker of anti-EGFR antibodies at present [47]. *HRAS* mutation could cause augmentation of phosphatidylinositide-3-kinase signaling [48] and also appeared in bladder and oropharyngeal cancer [49, 50]. Nevertheless, none of the mutations in the *RAS* gene family was a remarkable prognostic factor in CRC [46]. The PI3K protein encoded by *PIK3CA* was a lipid kinase that played a crucial role in promoting and regulating signal pathways relevant to cell proliferation, migration, apoptosis, and metabolism [51, 52]. *PIK3CA* mutation occurred 15–20% in colorectal cancer [53]. *PIK3CA* mutation contributed to the survival and proliferation of CRC stem cells, which induced chemotherapy resistance and poor prognosis [54], and reduced the hazard of peritoneal metastases [55]. PI3K upregulation was able to inhibit the apoptosis of CRC cells as well [56]. The expression level of PDE5A was upregulated after treatment with American ginseng and ginsenoside Rg3 in human CRC cells [57]. Significant association was found between PPARG variants and CRC [58]. PPARG might be the target of miR-34a and the potential therapeutic target of CRC [59]. Nonsteroidal anti-inflammatory drugs suppressed CRC stem cells via inhibiting PTGS2 and NOTCH/HES1 and activating PPARG [60]. The rs1590 variant of TGFBR1 might possess a significant association with CRC risk, and the hypomorphic variant TGFBR1 ∗ 6A affected migration and invasion in CRC cells [61, 62]. The miR-3191 promoted the migration and invasion by targeting TGFBR2 in CRC cells, and the miR-371∼373/TGFBR2/ID1 signaling axis might regulate the self-renewal of tumor-initiating cells and metastatic colonization as a novel mechanism [63, 64]. In summary, literature review supported HRAS, KRAS, PIK3CA, PDE5A, PPARG, TGFBR1, and TGFBR2 as crucial targets of HL-GJ against CRC and confirmed the reliability of key target screening via calculating topological parameters.

### 3.3. PPI Network of Herb-Compound-Compound Target-CRC Target-Other Human Proteins

**Figure 5:**
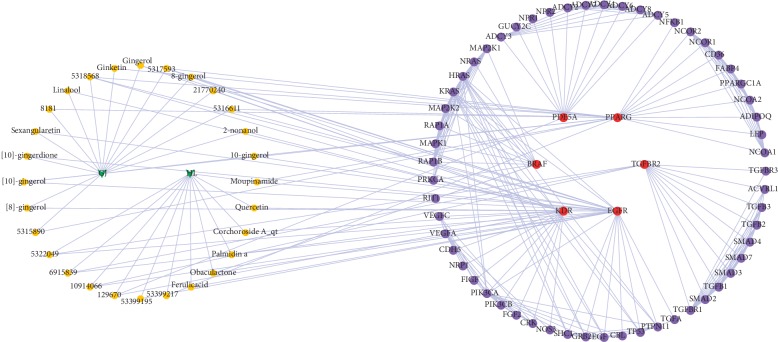
PPI network of herb-compound-compound target-CRC target-other human proteins traced the compounds acting on common targets and provided a straightforward reflection of the relationship from herb to compound to disease. Green arrow: herb; yellow octagon: chemical compound; red ellipse: common target between CRC and chemical compounds; purple ellipse: human protein directly or indirectly interacted with common target.

The network traced the compounds of HL-GJ acting on common targets between CRC and chemical compounds as shown in Figure 5, which covered 93 nodes (2 herb nodes, 28 compound nodes, 6 common target nodes, and 57 other human protein nodes) and 292 edges. The network provided a straightforward reflection of the relationship from herb to compound to disease. In order to identify more important compounds, the topological parameters of 28 compound nodes were calculated by NetworkAnalyzer ([Supplementary-material supplementary-material-1]). The median values of Degree, Betweenness Centrality, and Closeness Centrality were 2.54, 0.03, and 0.21, respectively. Nodes with “Degree >2.54,” “Betweenness Centrality >0.03,” and “Closeness Centrality >0.21” were regarded as major compounds of HL-GJ against CRC. Compounds satisfying requirements contained Gingerenone C, Isogingerenone B, 5,8-dihydroxy-2-(2-phenylethyl) Chromone, 2,3,4-trihydroxy-benzenepropanoic acid, 3,4-dihydroxyphenylethyl Alcohol Glucoside, 3-carboxy-4-hydroxy-phenoxy Glucoside, Moupinamide, and Obaculactone.

There have been few reports on the biological activities of diarylheptanoids containing Gingerenone C and Isogingerenone B, most of which exerted the effects of anti-inflammation, antioxidation, superoxide scavenging, and antihepatotoxicity [65, 66]. Gingerenone C has been reported to possess anti-inflammatory activity by inhibiting LPS-induced NO production in mouse RAW264.7 cells, which was isolated from rhizomes of *Curcuma kwangsiensis* [67]. 3,4-dihydroxyphenylethyl Alcohol Glucoside played antioxidant roles as a DPPH scavenger, hydroxyl radical scavenger, and superoxide anion radial scavenger by querying “Encyclopedia of Traditional Chinese Medicines: Molecular Structures, Pharmacological Activities, Natural Sources, and Applications.” Moupinamide showed anti-inflammatory activity via inhibiting NO generation in BV-2 induced by lipopolysaccharide with IC_50_ values of 8.17–18.73 *μ*M [68]. Obaculactone was assessed for oxidative burst inhibitory activity and for cytotoxicity against A549 lung carcinoma cells [69]. Obaculactone possessed anthelmintic, antiulcerative, inhibiting intestinal movement and other effects, referring to “Encyclopedia of Traditional Chinese Medicines-Molecular Structures, Pharmacological Activities, Natural Sources, and Applications.” The biological activities of the remaining compounds were rarely reported and needed to be further studied.

### 3.4. Gene Ontology Analysis

**Figure 6:**
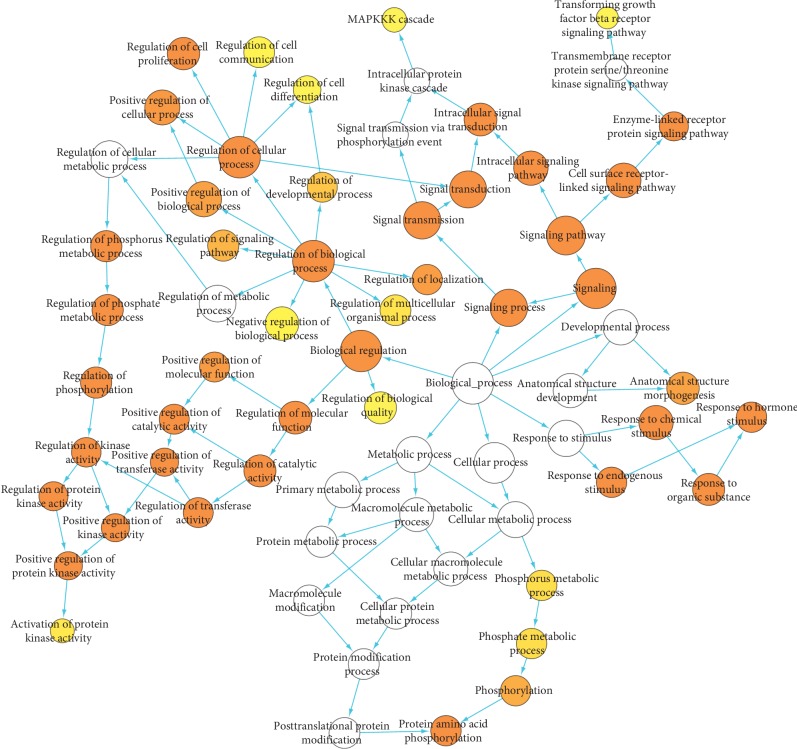
Gene Ontology (GO) biological process for PPI network. Yellow nodes indicate significant enrichment of biological process terms. The larger the yellow node, the more terms the enrichment. The darker the color, the smaller the *P* value (*P* < 0.001).

**Figure 7:**
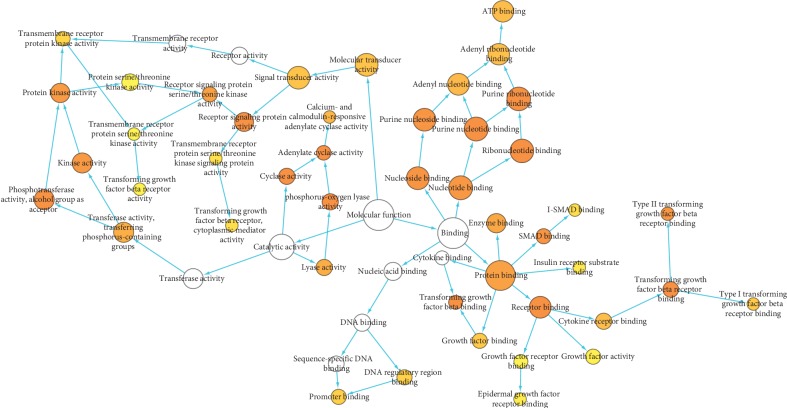
Gene Ontology (GO) molecular function for PPI network. Yellow nodes indicate significant enrichment of molecular function terms. The larger the yellow node, the more terms the enrichment. The darker the color, the smaller the *P* value (*P* < 0.001).

To illuminate the complex mechanisms of HL-GJ against CRC holistically, we conducted GO biological process and molecular function analysis for common targets and correlated other human protein targets. The main biological processes involved in HL-GJ against CRC are shown in Figure 6. The top 10 significantly enriched GO terms included signaling pathway, signaling, signal transduction, signal transmission, signaling process, regulation of phosphorylation, intracellular signaling pathway, cell surface receptor linked signaling pathway, regulation of phosphate metabolic process, and regulation of phosphorus metabolic process. The main molecular functions involved in HL-GJ against CRC are shown in Figure 7. The top 10 significantly enriched GO terms included phosphorus-oxygen lyase activity, cyclase activity, receptor signaling protein activity, transforming growth factor beta receptor binding, adenylate cyclase activity, receptor binding, purine nucleotide binding, ribonucleotide binding, purine ribonucleotide binding, and transforming growth factor beta binding. The yellow nodes represented GO terms with significant enrichment. The size of the node was consistent with the number of enriched terms, and the depth of the color was opposite of the *P* value. Detailed GO terms were listed in Tables [Supplementary-material supplementary-material-1] and [Supplementary-material supplementary-material-1], respectively.

Pathway enrichment analysis was executed based on Reactome database ([Supplementary-material supplementary-material-1]). There were mainly 162 pathways participating in HL-GJ against CRC. The top 10 significantly enriched pathways included DAP12 signaling, signaling by PDGF, signaling by EGFR, NGF signaling via TRKA from the plasma membrane, signaling by NGF, downstream signal transduction, DAP12 interactions, signaling by VEGF, signaling by FGFR3, and signaling by FGFR4. It was well to be reminded that the crucial targets calculated previously were contained in the hit genes of these pathways, which were highly correlated to CRC. DAP12 was an immunoreceptor tyrosine-based activation motif, bearing adapter molecules that transduced activation signals in NK and myeloid cells. DAP12-bound SYK autophosphorylated and phosphorylated the scaffolding molecule LAT, recruiting PI3K, PLC-gamma, GADS, SLP76, GRB2:SOS, and VAV, all of which resulted in the recruitment and activation of kinases AKT, CBL, and ERK, and rearrangement of the actin cytoskeleton finally leading to cellular activation [70]. As an immune antigen, DAP12 was expressed by tumor cells' “immune resistance” and avoided immune surveillance in CRC [71]. As important growth factors for normal tissue growth, division and blood vessel formation, PDGFs were correlated with invasion and metastasis and involved in angiogenesis mainly by targeting pericytes and vascular smooth muscle cells in CRC [72]. Anti-EGFR and anti-VEGF agents were now routinely incorporated into treating metastatic CRC, and the importance of signaling by EGFR and VEGF was self-evident [73]. For treating TrkA-overactive tumors, such as CRC and NGF, was praised as a “star” therapeutic target for decades to come [74]. NGF was demonstrated to strengthen the antiproliferation action of 5-FU on human CRC (HCT-116) cells and might reduce the dosage of 5-FU for CRC treatment [75]. It was reported that deregulation of signal transduction pathways played a critical role in oncogenesis of CRC and directly affected sensitivity to targeted therapies [76]. FGRFs were acknowledged oncogenes associated with a variety of cancers including CRC and were therefore attractive therapeutic targets [77]. Due to FGFR3-mediated essential survival signals in CRC, it might cause intrinsic resistance to Irinotecan, and the strong synergy was seen between the FGFR3 inhibitor and IRI [78]. The first specific inhibitor of FGFR4 was verified to restrain the proliferation of CRC cells, augment apoptosis rate, dispute cell cycle, and inhibit EMT, and might be a new targeted drug [79]. These results suggested that these main pathways might interact to produce the therapeutic efficacy of HL-GJ against CRC.

## 4. Conclusion

In this study, a systematical pharmacological approach was established to expound the active compounds, therapeutic targets, and pharmacological mechanisms of HL-GJ against CRC. Sixty-five constituent compounds of HL-GJ were summarized from TCMSP and TCMID, and their targets were predicted based on PharmMapper. One hundred and eighty-six CRC targets were collected from TTD and OMIM databases. Targets of CRC and chemical compounds were mapped to identify 6 common targets, and fifty-seven other human proteins directly or indirectly interacted with common targets were achieved from the String database. By network construction and topological parameter calculation, eight active compounds and seven crucial targets of HL-GJ against CRC were identified. Moreover, the biological processes, molecular functions and pathways regulated by HL-GJ treating CRC were systematically interpreted. This study provided a scientific and powerful mean to view the multiscale pharmacological mechanisms of HL-GJ against CRC from a systematical perspective.

## Data Availability

The data used to support the findings of this study are included within the supplementary information files

## Supplementary Materials

Supplementary Materials Table S1: chemical compounds of HL-GJ from TCMSP. Table S2: chemical compounds of HL-GJ from TCMID. Table S3: compounds in HL-GJ and corresponding targets. Table S4: CRC targets. Table S5: topological parameters for PPI Network. Table S6: topological parameters for compound nodes. Table S7: Gene Ontology (BP) biological process analysis. Table S8: Gene Ontology (MF) molecular function analysis. Table S9: pathway analysis.
